# Accuracy of Artificial Intelligence-Based Photographic Detection of Gingivitis

**DOI:** 10.1016/j.identj.2023.03.007

**Published:** 2023-04-26

**Authors:** Reinhard Chun Wang Chau, Guan-Hua Li, In Meei Tew, Khaing Myat Thu, Colman McGrath, Wai-Lun Lo, Wing-Kuen Ling, Richard Tai-Chiu Hsung, Walter Yu Hang Lam

**Affiliations:** a Faculty of Dentistry, The University of Hong Kong, Hong Kong Special Administrative Region, China; b School of Information Engineering, Guangdong University of Technology, Guangzhou, China; c Faculty of Dentistry, The National University of Malaysia, Kuala Lumpur, Malaysia; d Department of Computer Science, Hong Kong Chu Hai College, Hong Kong Special Administrative Region, China; e Musketeers Foundation Institute of Data Science, The University of Hong Kong, Hong Kong Special Administrative Region, China

**Keywords:** Gingivitis, Periodontal diseases, Community dentistry, Deep learning, Neural networks, computer, Artificial intelligence

## Abstract

**Objectives:**

Gingivitis is one of the most prevalent plaque-initiated dental diseases globally. It is challenging to maintain satisfactory plaque control without continuous professional advice. Artificial intelligence may be used to provide automated visual plaque control advice based on intraoral photographs.

**Methods:**

Frontal view intraoral photographs fulfilling selection criteria were collected. Along the gingival margin, the gingival conditions of individual sites were labelled as *healthy, diseased,* or *questionable*. Photographs were randomly assigned as training or validation datasets. Training datasets were input into a novel artificial intelligence system and its accuracy in detection of gingivitis including sensitivity, specificity, and mean intersection-over-union were analysed using validation dataset. The accuracy was reported according to STARD-2015 statement.

**Results:**

A total of 567 intraoral photographs were collected and labelled, of which 80% were used for training and 20% for validation. Regarding training datasets, there were total 113,745,208 pixels with 9,270,413; 5,711,027; and 4,596,612 pixels were labelled as *healthy, diseased,* and *questionable* respectively. Regarding validation datasets, there were 28,319,607 pixels with 1,732,031; 1,866,104; and 1,116,493 pixels were labelled as *healthy, diseased*, and *questionable*, respectively. AI correctly predicted 1,114,623 *healthy* and 1,183,718 *diseased* pixels with sensitivity of 0.92 and specificity of 0.94. The mean intersection-over-union of the system was 0.60 and above the commonly accepted threshold of 0.50.

**Conclusions:**

Artificial intelligence could identify specific sites with and without gingival inflammation, with high sensitivity and high specificity that are on par with visual examination by human dentist. This system may be used for monitoring of the effectiveness of patients’ plaque control.

## Introduction

Periodontal disease is a chronic inflammatory disease that affects the periodontium and is categorised into gingivitis and periodontitis with reversible and irreversible tissue damages, respectively.^1^^,^^2^ It is one of the most prominent oral diseases, accounting for a significant amount of global public health burden every year, as well as 21% of global productivity loss, equivalent to USD 38.85 billion.[3], [4], [5] The prevalence of periodontal disease is estimated to be more than 50% worldwide, and nearly one-third of them are severe cases, that is, clinical attachment loss of more than 5 mm and bone loss of more than 30%, according to the World Health Organization.^3^^,^[6], [7], [8]

Periodontal disease is caused by accumulation of plaque biofilm along the gingival margin, resulting in localised gingival inflammation and host responses.[9], [10], [11], [12], [13] An early stage of periodontal disease, gingivitis, may be reversed by removal of plaque, and the progress to later stages of periodontitis may be halted.^14^

Furthermore, the development of periodontal disease is not consistent amongst all teeth and sites, and site predilections, i.e., site-specific, have been observed.^15^^,^^16^ For proper self-care or professional care, understanding and evaluations of clinical signs of individual sites are crucial.^17^ The clinical signs of gingivitis are inflammation-related and are a result of host response to dental plaque. As inflammation occurs at the gingival margin, redness (ie, change in colour); swelling (ie, change in volume); and loss of stippling appearance as loss of gingival fiber attachment (ie, change in surface characteristics) are observed, due to increase in blood flow (redness) and leakage of tissue fluid from blood vessels into the tissues (swelling).^18^^,^^19^ These changes are generally assessed visually by dentists, and patients may not be aware of the disease progression due to its chronic nature and lack of acute symptoms.^16^^,^^20^^,^^21^

Effective self-care plaque control measures such as tooth brushing and interdental cleaning are keys to periodontal disease prevention and control.^22^ Studies have revealed that frequent dental appointments are expensive yet ineffective in achieving sustained satisfactory plaque control at specific sites despite significant resources being committed to motivate and reinforce patients’ oral hygiene and plaque control measures.^3^^,^^23^^,^^24^

Artificial intelligence (AI) may provide a solution to this persistent clinical problem. The application of AI in various areas of dentistry has been gaining traction amongst the dental communities in recent years under name of automated digital dentistry and Dentistry 4.0.^25^ There are many clinical applications of AI in dentistry, from analysis of 2D radiography to 3D crown reconstruction, and AI has been utilised in detection of gingivitis from intraoral photographs.[26], [27], [28], [29], [30], [31], [32], [33], [34], [35], [36], [37] However, according to a recent literature review, there seems to be a lack of agreement in assessing the accuracy of prediction of gingivitis by AI systems, though 0.90 or above is regarded as excellent diagnostic accuracy for a general test.^30^^,^^38^^,^^39^ For an AI system to be used clinically for predicting gingivitis, it should have high sensitivity, that is, report *diseased* for any site where there is gingivitis, and high specificity, that is, report *healthy* for any site where there is no gingivitis. These parameters are one of the commonly used medical and dental diagnostic performance metrics^40^ and are proposed to measure the accuracy of AI prediction in this study.^41^

There are several network architectures that are currently used to detect gingivitis from intraoral photographs with accuracy ranging from 0.47 to 0.83, with 1.00 as the highest accuracy.^30^^,^[35], [36], [37]^,^^34^^,^^42^^,^^43^ The accuracy of any diagnostic system for clinical use should be as high as possible, and accuracy of 0.90 or above should be targeted for clinical use.^38^^,^^39^^,^^44^^,^^45^

**Fig. 1:**
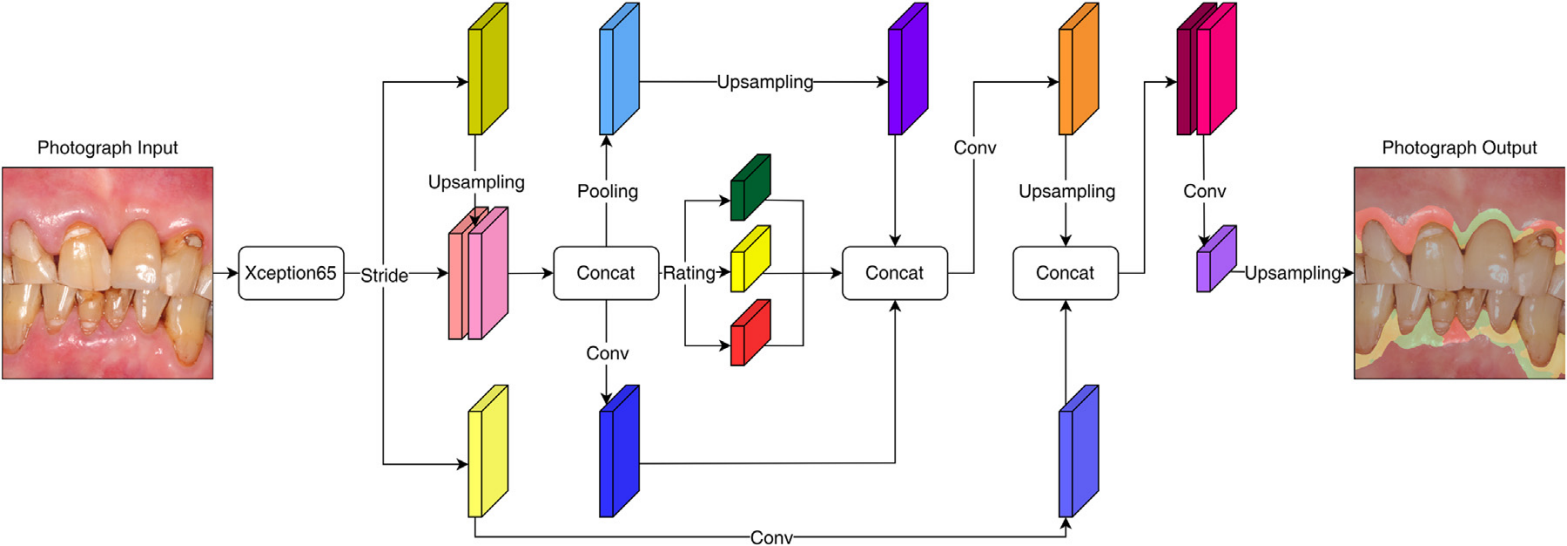
Illustration of architecture of DeepLabv3+ neural networks in this study.

In this study, DeepLabv3+ built on Keras (v2.12, Google LLC) with TensorFlow 2 (v2.9, Google LLC) was adopted. This neural network was highly transferable and offered multiple pretrained checkpoints to facilitate learning of the datasets (Figure 1).[46], [47], [48], [49] Xception (v1.0, Google LLC) and MobileNetV2 (v1.0, Google LLC) were adopted as the backbone. Xception models used depth-wise separable convolutions with fewer connections and lighter model (ie, faster), and MobileNet models utilised the same convolutions with smaller model size and complexity, making it easier to construct.^50^^,^^51^

**Fig. 2:**
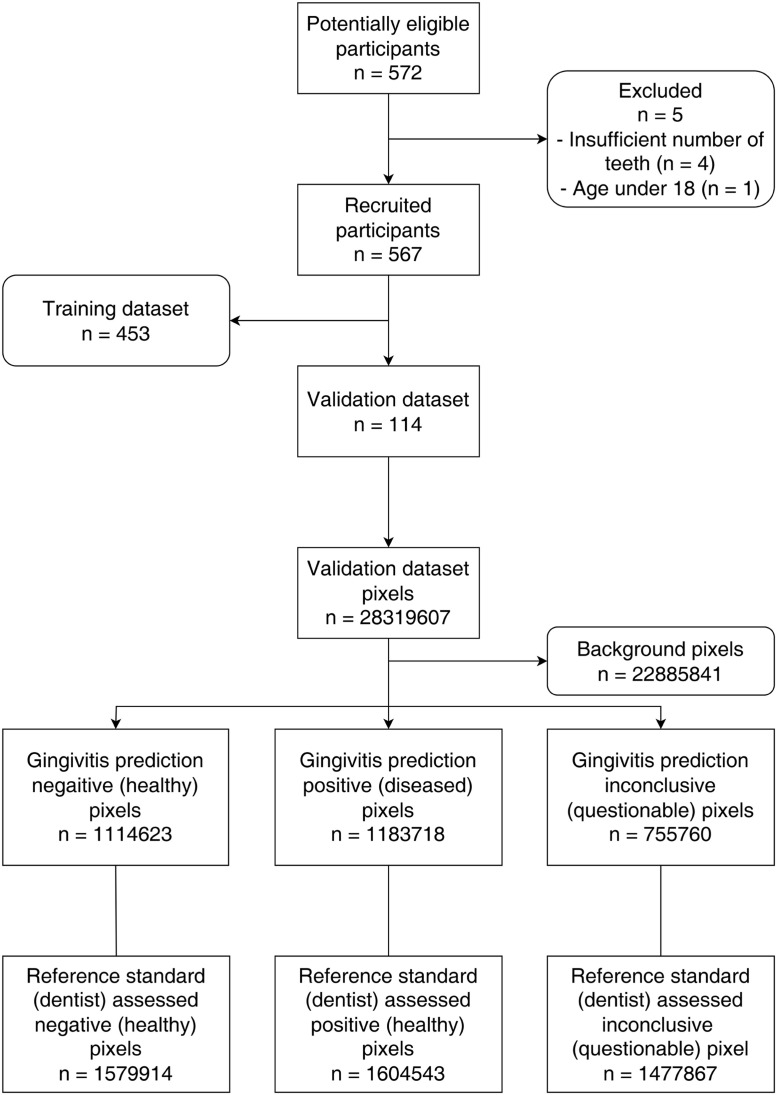
STARD-2015 flow diagram of this study.

The objective of this study was to develop and to validate a novel AI system that can be used to diagnose gingivitis on intraoral photographs with accuracy at or above 0.90. The hypothesis of this study was that a novel AI system built with DeepLabv3+, after training with adequate number of intraoral photographs, would be able to predict the gingival health status with accuracy, in terms of sensitivity and specificity, at or above 0.90. This study was reported in format according to the Standards for Reporting Diagnostic Accuracy (STARD) 2015 statement (Figure 2).^52^

## Methods

This study was approved by the Institutional Review Board of the University of Hong Kong/Hospital Authority Hong Kong West Cluster (HKU/HA HKW IRB), Hong Kong Special Administrative Region, China (reference numbers: UW 20-230 and UW 21-447), and the Research, Ethics/Safety Sub-Committee (RESS) of Hong Kong Chu Hai College, Hong Kong Special Administrative Region, China (reference number: RESS/2022/06/006). This study was a prospective study, and data collection was planned before the execution of this study.

### Data collection

Consecutive participants were recruited amongst patients attending the Comprehensive Dental Clinic of the University Dental Hospital from 2020 to 2022 according to the selection criteria (Table 1). Informed consent was obtained from all participants. Frontal-view intraoral photographs were taken using a digital single lens reflex (SLR) camera (EOS 700D, Canon) with a macro lens (EF 100mm f/2.8, Canon) and a ring flash (Marco Ring Lite MR-14EX, Canon). The sample size used for training the AI system was based on a recent study on using AI to detect periodontitis, which featured around 450 training datasets.^53^

**Table 1:** Inclusion and exclusion criteria of study participants.

Inclusion criteria	Participants who are Chinese and aged 18 or older
	Participants who are able to give consent
	Participants who have 5 or more anterior teeth
	Participants who have adequate mouth opening for visualisation of at least 3 mm gingival tissue from maxillary and mandibular gingival margins
	Participants who are able to attend dental appointment and hold still during taking intraoral photograph
Exclusion criteria	Participants who have non-plaque-related oral mucosal diseases that preclude the use of mirror retractors

### Data preparation

The gingival conditions of the collected intraoral photographs were labelled by a calibrated assessor, who was a dentist and based on visual assessment on a computer monitor (P2419H 23.8” W-LED monitor, Dell). The areas of interest within each frontal photograph were the gingival margin and around 3 mm gingival tissues apical to the margin. These areas were classified into 1 of 3 categories: *healthy, diseased*, or *questionable,* based on a screening instrument, Oral Health Assessment Tools (OHATs),[54], [55], [56] where the definitions were as follows:

- Healthy: pink, smooth, no bleeding
- Questionable: red, rough, swollen
- Diseased: white/red patches, generalised redness, ulcers, swollen, bleeding

Unlabelled areas were classified in the system as *background*, making a total of 4 classifications. One week later, 10% of all photographs were labelled again by same assessor to measure the intra-assessor reliability in diagnosis of gingival conditions *healthy, diseased,* or *questionable*.^57^^,^^58^ The kappa value of the assessor was measured.

Around 450 photographs were randomly designated as training datasets by randomisation table, and the rest of the photographs were designated as validation datasets.

Photographs of the training datasets were augmented by cropping, rotating, or flipping randomly to enhance the training quality.^59^

### Training and validation

Photographs from the training datasets were input into the AI system for training. After training, the AI system was then instructed to diagnose the gingival status of intraoral photographs of the validation datasets. Both the training and validation processes were performed on a Linux system powered by a graphic card of NVIDIA GeForce RTX 3090. The batch number was set as 4, which is the number of classifications, and the number of training iterations was set to be 30,000, a common iteration number to train 2-dimension AI systems.^60^^,^^61^

### Measurements

The performance of the AI system was measured by true-positive rate, true-negative rate, false-positive rate, and false-negative rate. True-positive rate was the outcome where the AI correctly detected the diseased status, and true-negative rate was the outcome where the AI correctly detected the healthy status. False-positive rate and false-negative rate were the outcomes where AI treated healthy sites as diseased and diseased sites as healthy, respectively. Sensitivity and specificity were calculated based on the following formula:

$$\begin{array}{c} Sensitivity=TruePositive/\left(TruePositive+FalseNegative\right)\\ Specificity=TrueNegative/\left(TrueNegative+FalsePositive\right) \end{array}$$

Mean intersection-over-union, a ratio of true predictions (positive and negative) against the ground truth (actual health status), was a wide-adapted performance metric for segmentation models in field of artificial intelligence and was calculated by dividing the sum of 4 intersection-over-unions of *healthy, diseased, questionable*, and *background* by 4.^62^ Intersection-over-union of each category was calculated by the following formula:

Intersection−Over−Union=(α∩β)/(α∪β)

where *α* was the dataset of diagnosis by dentist and *β* was the dataset of prediction by the AI system. In mathematics, the symbol ∪ represents the union of 2 sets, whilst ∩ represents the intersection of the sets.

Accuracy ranged from 0.00 to 1.00, and 1.00 was considered to be the maximum accuracy.^63^ The common threshold for acceptable prediction was 0.50.^64^

## Results

In all, 572 potential participants were screened according to the study criteria. Four were rejected due to insufficient number of anterior teeth, and one was rejected due to age younger than 18 years. The number of recruited study participants was 567.

A total of 567 frontal-view intraoral photographs were taken from the study participants. Amongst the collected photographs, around 80% of the total (n = 453) were designated as training datasets, and the rest (n = 114) were designated as validation datasets.

The training datasets consisted of 113,745,208 pixels in total, with 9,270,413; 5,711,027; and 4,596,612 pixels labelled as *healthy, diseased*, and *questionable*, respectively. The validation datasets consisted of 28,319,607 pixels in total, with 1,579,914; 1,604,543; and 1,477,867 pixels labelled as *healthy, diseased*, and *questionable*, respectively. The assessor had a kappa value 0.92 over 2 attempts of labelling, which indicated high reliability.

**Fig. 3:**
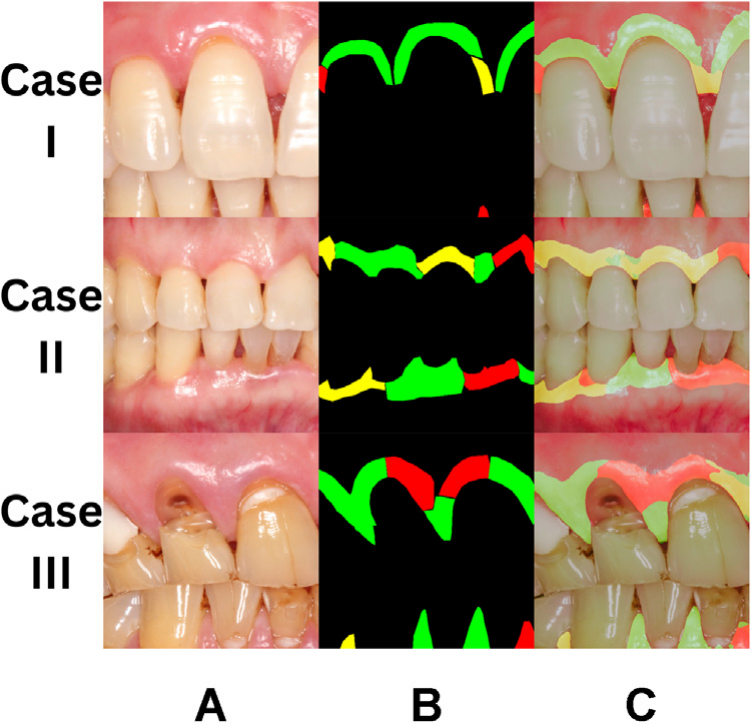
Selected detection results of the validation set using the adopted segmentation model. A, Input intraoral photograph. B, Ground truth (health status) labelled by calibrated dentist. C, Detection results: green = healthy, red = diseased, yellow = questionable.

The AI system was then validated using intraoral photographs from the validation datasets, and results are presented in Figure 3. AI correctly predicted 1,114,623 *healthy* and 1,183,718 *diseased* pixels (Table 2), with a sensitivity of 0.92 and a specificity of 0.94. The mean intersection-over-union was 0.60.

**Table 2:** Predictions of the AI system compared to the diagnosis of a calibrated dentist.

		Predictions of the AI system (pixels)			
		Predicted as healthy	Predicted as diseased	Predicted as questionable	Predicted as background
Diagnosis of dentist (pixels)	Diagnosed healthy	1,114,623	72,048	140,694	252,549
	Diagnosed diseased	93,017	1,183,718	76,597	251,211
	Diagnosed questionable	248,694	258,035	755,760	215,378
	Background	275,697	352,303	143,442	22,885,841

## Discussion

The results of this study supported the hypothesis that a novel AI system built with DeepLabv3+, after training with adequate number of intraoral photographs, would be able to predict the gingival health status with accuracy, in terms of sensitivity and specificity, at or above 0.90. The novel AI system was able to identify specific sites with and without gingival inflammation with sensitivity and specificity that were almost on par with human dentists, which is one of the current methods used to detect gingival inflammation clinically.^39^^,^^40^^,^^64^ The result was encouraging and supported the use of AI in detection of gingivitis on intraoral photographs.

The AI system still had limitations and needed further development. Because the training was based on Chinese participants, the resulting system may work better on Chinese individuals compared with other ethnicities including White, Latino, and Black, though determining whether such a difference exists still needs further examination. Also, there was no evidence yet to suggest it would retain the same performance when it was applied to patients with various local and systemic modifying factors.^14^ Further studies into applications of this novel AI system in gingival inflammation detection would also be needed to further improve the accuracy of the system, with a goal of achieving superior performance as a periodontist. Moreover, clinical examination of the gingival conditions by probing might reduce the area with a questionable diagnosis and provide more robust gingival conditions for AI to learn. In addition, the performance of this system in clinical settings should be investigated with a longitudinal clinical trial design. Apart from the diagnosis of gingival conditions, the consistency of the outline of labeled areas should be addressed in the reliability test of the assessor.

When a population has a high prevalence of a particular disease such as gingivitis, it is expected that its diagnostic tests usually have high sensitivity, that is, a positive result when there is a disease, and low specificity, that is, a negative result when there is no disease. This is because it is easier for a diagnostic test to detect a disease when it has high prevalence and vice versa. However, gingivitis is a site-specific disease, and healthy sites may be found in patients with gingivitis. Therefore, similar numbers of healthy and diseased pixels as well as similar levels of sensitivity and specificity are found in this study.

With training datasets in larger quantities as well as in decreased diversity, the training outcomes may be further improved. However, room for improvement may be limited because the accuracy of this system was already above 0.90. Future studies would likely pave the way for applications of such AI systems in periodontology and, in a greater aspect, prevention and control of periodontal disease in communities.

## Conclusions

AI is able to identify specific sites with and without gingival inflammation with high sensitivity and high specificity. Further investigation and training are required for possible improvements and clinical applications.

## Conflict of interest

None disclosed.

## Appendix Supplementary materials

Image, application 1

Image, application 2
