# Supplementary material for: Accuracy of Artificial Intelligence-Based Photographic Detection of Gingivitis
Source: Int Dent J. 2023 Apr 26;73(5):724–30. doi: 10.1016/j.identj.2023.03.007 (PMC10509417; doi:10.1016/j.identj.2023.03.007)
Supplement: Supplementary file 2 [file mmc2.docx]

11 January 2023

Professor Lakshman Samaranayake

Editor-in-Chief

International Dental Journal

Dear Editor-in-Chief,

I understand it is the policy of the Journal to have a maximum of 6 authors. However, the submitted manuscript “**Artificial Intelligence-based Photographic Detection of Gingival Inflammation**” involves members from multi-disciplines such as subject recruitment and collection of data, development of artificial intelligence as well as validation and technical assessment. Therefore, I would like to list all contributing team members to recognize their efforts and contributions in this study.

Dr. Reinhard Chau, Dr. Richard Hsung and I myself, as a team, have carried out significant amounts of research works throughout the study. Mr. Guanhua Li, Dr. In Meei Tew and Dr. Khaing Myat Thu have provided valuable expertise and resources for the project. Prof. Wai-Lun Lo, Prof. Wing-Kuen Ling and Prof. Colman McGrath have given significant support for the project, especially in terms of study designs and technical supports. The study may not happen without them. I believe it is only right to include everyone on the authorship.

I have attached with this letter a CREDIT author statement for your reference. We look forward to receiving a favourable response from you. Thank you for your kind consideration.

Yours Sincerely,

Walter Lam (Corresponding Author)

Email: retlaw@hku.hk

# CRediT author statement

**Reinhard Chun Wang Chau:** Validation, Formal analysis, Investigation, Resources, Data Curation, Writing – Original Draft, Writing – Review & Editing, Visualization

**Guan-Hua Li:** Conceptualization, Methodology, Software, Validation, Formal analysis, Writing – Review & Editing
**In Meei Tew:** Resources, Data Curation

**Khaing Myat Thu:** Resources, Data Curation

**Colman McGrath:** Writing – Review & Editing, Supervision

**Wai-Lun Lo:** Software, Supervision

**Wing- Kuen Ling:** Conceptualization, Methodology, Software, Writing – Review & Editing, Supervision

**Richard Tai-Chiu Hsung**: Conceptualization, Methodology, Software, Validation, Writing – Review & Editing, Supervision, Project administration, Funding acquisition

**Walter Yu Hang Lam:** Conceptualization, Methodology, Validation, Writing – Review & Editing, Supervision, Project administration, Funding acquisition
